# Antibiofilm and Anti-Inflammatory Activities of* Houttuynia cordata* Decoction for Oral Care

**DOI:** 10.1155/2017/2850947

**Published:** 2017-10-16

**Authors:** Yasuko Sekita, Keiji Murakami, Hiromichi Yumoto, Kouji Hirao, Takashi Amoh, Natsumi Fujiwara, Katsuhiko Hirota, Hideki Fujii, Takashi Matsuo, Yoichiro Miyake, Yoshiki Kashiwada

**Affiliations:** ^1^Department of Pharmacognosy, Institute of Biomedical Sciences, Tokushima University Graduate School, Tokushima 770-8505, Japan; ^2^Department of Oral Microbiology, Institute of Biomedical Sciences, Tokushima University Graduate School, Tokushima 770-8504, Japan; ^3^Department of Conservative Dentistry, Institute of Biomedical Sciences, Tokushima University Graduate School, Tokushima 770-8504, Japan

## Abstract

Dental biofilms that form in the oral cavity play a critical role in the pathogenesis of several infectious oral diseases, including dental caries, periodontal disease, and oral candidiasis.* Houttuynia cordata* (HC, Saururaceae) is a widely used traditional medicine, for both internal and external application. A decoction of dried HC leaves (dHC) has long been consumed as a health-promoting herbal tea in Japan. We have recently reported that a water solution of HC poultice ethanol extract (wHCP) exerts antimicrobial and antibiofilm effects against several important oral pathogens. It also exhibits anti-inflammatory effects on human keratinocytes. In our current study, we examined the effects of dHC on infectious oral pathogens and inflammation. Our results demonstrated that dHC exerts moderate antimicrobial effects against methicillin-resistant* Staphylococcus aureus* (MRSA) and other oral microorganisms. dHC also exhibited antibiofilm effects against MRSA,* Fusobacterium nucleatum* (involved in dental plaque formation), and* Candida albicans* and inhibitory effects on interleukin-8, CCL20, IP-10, and GRO*α* productions by human oral keratinocytes stimulated by* Porphyromonas gingivalis* lipopolysaccharide (a cause of periodontal disease), without cytotoxic effects. This suggests that dHC exhibits multiple activities in microorganisms and host cells. dHC can be easily prepared and may be effective in preventing infectious oral diseases.

## 1. Introduction

Dental biofilms that form in the oral cavity play a critical role in the pathogenesis of numerous infectious oral diseases, including periodontal disease. This can be due to the absorption of antimicrobial and antiseptic drugs and development of resistance to host immune cells [1–3]. Predominant fungi such as* Candida albicans* in the oral cavity can also contribute to the development of infectious oral diseases ranging from denture stomatitis [4, 5] to life-threatening invasive infections, including aspiration pneumonia. This is particularly apparent in immunocompromised and elderly patients [6–8]. We have previously reported a higher prevalence of* Candida* spp.,* Pseudomonas aeruginosa*, and* Staphylococcus* spp. in the oropharyngeal microflora of patients with cerebrovascular infarction and dysphagia [6]. Reducing adherence and biofilm formation by oral microorganisms can contribute to the prevention of chronic oral infections that may lead to potentially severe, systemic opportunistic diseases, particularly in the elderly [9, 10].

Oral keratinocytes play an important role as the first physical barrier to bacterial invasion by organizing the local innate immune system against colonizing microorganisms. They also secrete several proinflammatory mediators (i.e., chemokines and cytokines) in response to various stimuli, including microbial infections and chemical or thermal irritations [11–13]. These mediators ultimately cause periodontal inflammation. Lipopolysaccharide (LPS) from Gram-negative bacteria, such as the important periodontal pathogen* Porphyromonas gingivalis*, upregulates the production of various proinflammatory mediators via signal cascades in the gingival epithelium. These include interleukin-8 (IL-8), CCL20, IFN-*γ*-inducible protein 10 (IP-10), and growth related oncogene-*α* (GRO*α*) [14–22]. Therefore, the development of oral care products that reduce biofilm formation and subsequent proinflammatory responses is essential for improving health and preventing disease.

Previous studies have demonstrated that extracts from medicinal plants exhibit various pharmacological activities including antimicrobial effects [23–26], antiadherence effects against oral microorganisms [27–29], and anti-inflammatory effects [30–33].* Houttuynia cordata* Thunb. (HC, Saururaceae) is widely used as a traditional medicine, both internally and externally [34]. However, evidence to indicate that HC extract exerts pharmacological effects against oral microorganisms is limited. We have focused on identifying and characterizing any activity of HC against infectious diseases caused by oral microorganisms. We have recently reported that a HC poultice ethanol extract (eHCP) exerted antibacterial effects against cutaneous infection-related bacteria and anti-inflammatory effects on human keratinocytes [34]. However, caution is required when suggesting that eHCP could be applied to oral care because ethanol found in mouthwashes has been suggested to increase the risk of oral cancer [35].

Fortunately, we have also shown that a water solution of eHCP (wHCP) exhibits antimicrobial and antibiofilm effects on oral microorganisms and anti-inflammatory effects on oral keratinocytes [36]. A decoction of dried HC leaves (dHC), commonly consumed as a health-promoting herbal tea in Japan, is simpler to prepare than wHCP. We therefore examined the antimicrobial and antibiofilm effects of dHC on several important infectious oral pathogens and investigated the anti-inflammatory effects of dHC on* P*.* gingivalis* LPS-stimulated human oral keratinocytes.

## 2. Material and Methods

### 2.1. Plant Materials and Sample Preparation

HC used in this study was collected in Kochi City and identified by Dr. K. Fujikawa (the Kochi Prefectural Makino Botanical Garden, Kochi, Japan). Voucher specimens (FOS-007536, FOS-007537, and FOS-010389) were also deposited here.

### 2.2. Preparation of dHC

dHC was prepared as follows: 3 g of dried HC leaves was decocted with 130 mL of sterile purified water at 90–95°C for 30 min (EK-SA 10, ZOJIRUSHI, Osaka, Japan). The decoction was then centrifuged for 15 min at 1,500 ×g. After centrifugation, the clear supernatant layer was filtered through a 0.45-*μ*m filter and stored at 4°C until being assayed.

### 2.3. Flavonoid Glycosides

Quercitrin and rutin were purchased from Sigma-Aldrich (St. Louis, MO) and Tokyo Chemical Industry Co., Ltd. (Tokyo, Japan), respectively. Isoquercitrin and hyperin were isolated from the aerial parts of Hypericum sikokumountanum [37]. Flavonoid glycosides were dissolved in dimethyl sulfoxide (DMSO, nacalai tesque, Kyoto, Japan).

### 2.4. Bacterial Strains and Growth Conditions

The bacterial strains used in this study are shown in Table 1.* P. aeruginosa* was grown in Muller-Hinton broth (Becton Dickinson, Sparks, MD, USA) that was supplemented with 50 *μ*g/mL CaCl_2_ and 25 *μ*g/mL MgCl_2_. Methicillin-resistant* Staphylococcus aureus* (MRSA) strains were grown in Muller-Hinton broth supplemented with 25 *μ*g/mL CaCl_2_, 12.5 *μ*g/mL MgCl_2_, and 2% NaCl [38].* Streptococcus* spp. were grown anaerobically in brain heart infusion (Becton Dickinson).* Fusobacterium nucleatum* and* P. gingivalis* were grown anaerobically in brain heart infusion supplemented with 5 *μ*g/mL hemin and 0.5 *μ*g/mL menadione.* Aggregatibacter actinomycetemcomitans* was grown anaerobically in Todd Hewitt Broth (OXOID Ltd., Hampshire, UK).* C. albicans* was grown in Sabouraud dextrose medium composed of 10 g/L peptone and 40 g/L glucose. For biofilm formation assays, trypticase soy broth (Becton Dickinson) supplemented with 5 *μ*g/mL hemin and 0.5 *μ*g/mL menadione, trypticase soy broth supplemented with 0.3% glucose, and yeast nitrogen base medium at pH 7 containing 2.5 mmol/L N-acetylglucosamine [39] were used for* F*.* nucleatum*, MRSA-T31, and* C*.* albicans*, respectively.

### 2.5. Susceptibility Assay

The minimum inhibitory concentration (MIC) of dHC was assessed using a microbial broth dilution method. Approximately 10^6^ colony-forming units (CFU)/mL of each bacterial culture were inoculated into 100 *μ*L of medium containing a twofold serial dilution of dHC in 96-well plates (TPP, Trasadingen, Switzerland) and incubated either anaerobically (for* Streptococcus* spp.,* A. actinomycetemcomitans*,* F. nucleatum*, and* P. gingivalis*) or aerobically (for MRSA T31, MRSA COL,* P. aeruginosa*, and* C. albicans*) at 37°C for 20 or 48 h. The MIC was defined as the lowest concentration that showed no bacterial growth.

### 2.6. Biofilm Formation Assay

A crystal violet biofilm assay was performed to quantify the biofilm mass as previously described [40]. A 2-*μ*L (10^7^ CFU/mL) sample of MRSA T31 or* C. albicans* CAD1 in the stationary phase or a 5-*μ*L (10^7^ CFU/mL) sample of* F. nucleatum* JCM8532 in the stationary phase was transferred into a 96-well plate (Cellstar, Greiner Bio-One, Frickenhausen, Germany) from the primary 150 *μ*L suspensions of broth or media. dHC was then added to a final concentration of 10%. Quercitrin, isoquercitrin, hyperin, and rutin were added to a final concentration of 200 *μ*g/mL. Bacterial suspensions were incubated either anaerobically (for* F. nucleatum*) at 37°C for 24 h or aerobically (for MRSA T31 and* C. albicans*) at 37°C for 6 and 24 h. For the positive control, 2 or 1 *μ*g/mL of cetylpyridinium chloride (CPC) (Tokyo Chemical Industry Co., Ltd., Tokyo, Japan) was used [41]. For the negative control, 10% distilled H_2_O was used. After incubation, any biofilms that formed were washed with purified water twice without disturbing the adherent biofilm. They were then stained with 150 *μ*L of 0.1% crystal violet at 25°C for 10 min. Excess staining was removed by gentle washing with purified water. After drying, stained biofilms were extracted from each well by adding 150 *μ*L of ethanol, and the absorbance of the extract from the stained biofilm was measured at 595 nm using a microplate reader (model 680; Bio-Rad Laboratories, Hercules, CA, USA).

### 2.7. Cell Culture

RT-7 cells, an immortalized human keratinocyte cell line kindly provided by Dr. Kamata (Hiroshima University, Hiroshima, Japan) [42], were cultured in Keratinocyte-SFM (Gibco BRL, Gaithersburg, MD, USA) supplemented with 100 U/mL penicillin and 100 *μ*g/mL streptomycin (Gibco BRL), at 37°C in a water-saturated atmosphere of 95% air and 5% CO_2_. Confluent monolayers were cultured with 1 *μ*g/mL* P. gingivalis* LPS (InvivoGen, San Diego, CA, USA), with and without the addition of 1% dHC or 50 *μ*g/mL of quercitrin, isoquercitrin, hyperin, or rutin.

### 2.8. Lactate Dehydrogenase (LDH) Cytotoxicity Assay

The effects of dHC on cell cytotoxicity were assessed using a LDH assay. Confluent RT-7 cell monolayers in a 24-well plate were cultured in Keratinocyte-SFM medium supplemented with 10% and 1% dHC at 37°C for 24 h in a water-saturated atmosphere of 95% air and 5% CO_2_. As a positive control, RT-7 cells were treated with 0.1% Triton X-100 at 25°C for 10 min. In the cytotoxicity assay, the levels of LDH released into the recovered cell culture supernatants were measured using an LDH cytotoxicity assay kit (Cayman Chemical, Ann Arbor, MI, USA) following the manufacturer's instructions. Absorbance was measured at 490 nm using a microplate reader (Bio-Rad Laboratories).

### 2.9. Enzyme-Linked Immunosorbent Assay

Enzyme-linked immunosorbent assay (ELISA) kits were used to quantify the levels of IL-8, CCL20, IP-10, and GRO*α* (R&D Systems, Minneapolis, MN, USA) in cell culture supernatants.

### 2.10. Statistical Analysis

All statistical analyses were performed using an unpaired Student's *t*-test. Differences were considered significant when the probability value was less than 5%  (^*∗*^*P* < 0.05).

## 3. Results

### 3.1. Moderate Antimicrobial Effects of dHC

We examined the antimicrobial effects of dHC against several oral microorganisms. As shown in Table 2, dHC exerted a moderate antimicrobial effect against MRSA T31, MRSA COL,* S. intermedius*,* S. mitis*,* F. nucleatum*, and* P. gingivalis* (MIC; 375–1500 *μ*g/mL).

### 3.2. Antibiofilm Effects of dHC

**Figure 1:**
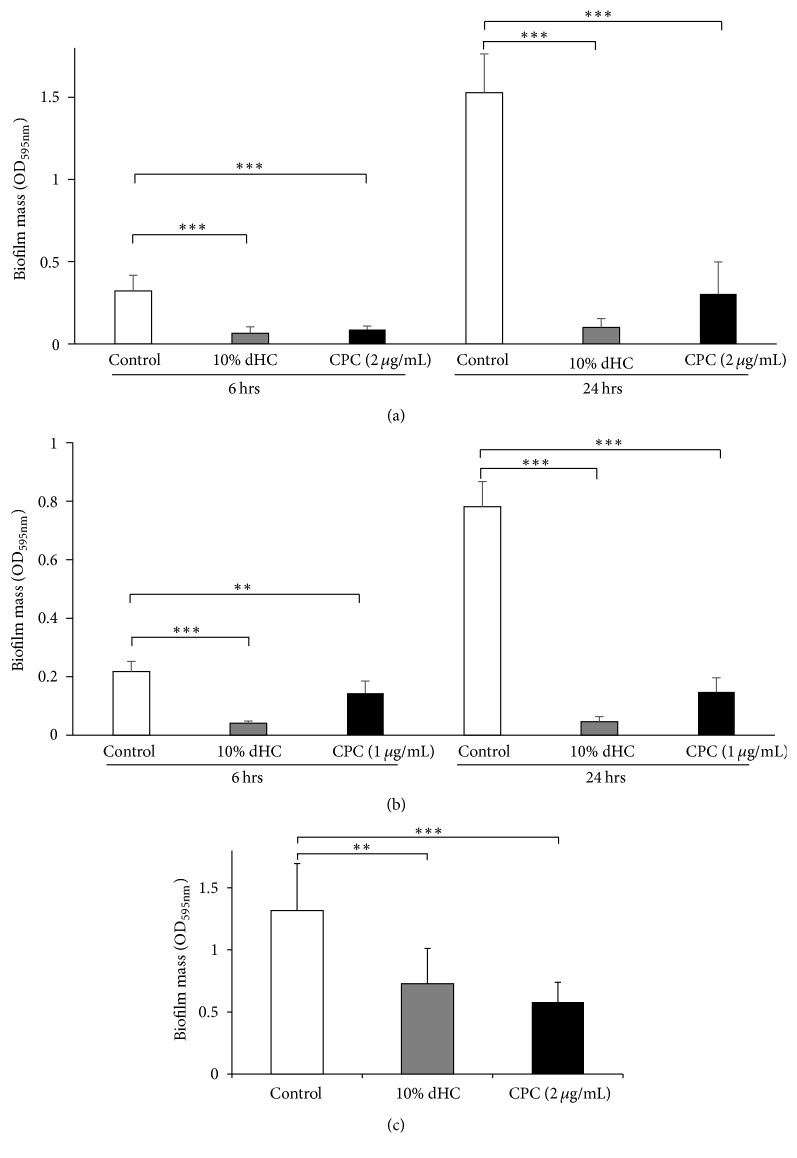
*Antibiofilm effects of dHC on MRSA T31, C. albicans, and F. nucleatum*. Antibiofilm effects of a water decoction of* Houttuynia cordata* (dHC) on biofilm formation by MRSA T31 (a) and CAD1 (b) at 6 or 24 h. Antibiofilm effects of dHC on biofilm formation by* F*.* nucleatum* JCM8532 (c) at 24 h. As a positive control, 2 *μ*g/mL (for MRSA T31,* F*.* nucleatum*) or 1 *μ*g/mL (for CAD1) of cetylpyridinium chloride (CPC) was used. A negative control of 10% distilled H_2_O was used. ^*∗∗∗*^Significant differences between the indicated groups at *p* < 0.001. ^*∗∗*^Significant differences between the indicated groups at *p* < 0.01 using a Student's *t*-test (*n* = 8).

We next investigated whether dHC had any antibiofilm effects. In this experiment, we used a culture of the MRSA T31 clinical isolate. This strain was selected because MRSA T31 exhibits increased biofilm formation compared to MRSA COL (data not shown). We also examined* F. nucleatum* and* C*.* albicans*. Both of these species form biofilms in the human oral cavity, including on denture surfaces [43, 44]. A biofilm formation assay at 6 and 24 h revealed that 10% dHC significantly inhibited biofilm formation by MRSA T31 (Figure 1(a)) and* C. albicans* (Figure 1(b)). We also showed that 10% dHC significantly inhibited 24-h biofilm formation by* F. nucleatum* (Figure 1(c)). In each experiment, 10% dHC did not affect the growth of these microorganisms (data not shown). These results revealed that dHC exhibits an antibiofilm effect against MRSA T31,* F. nucleatum*, and* C. albicans*. However, dHC did not exert any antibiofilm effects against* S. mutans* MT8148, a cause of dental caries (data not shown). The MICs of dHC against MRSA T31,* F. nucleatum*, and* C*.* albicans* were 5% (750 *μ*g/mL), 2.5% (375 *μ*g/mL), and >10% (>1500 *μ*g/mL), respectively. In the biofilm formation assay, bacterial abundance was approximately 100-fold higher than that in the MIC assay. We also observed that bacteria grew despite high concentrations of dHC.

### 3.3. No Cytotoxic Effects of dHC on Oral Keratinocytes

**Figure 2:**
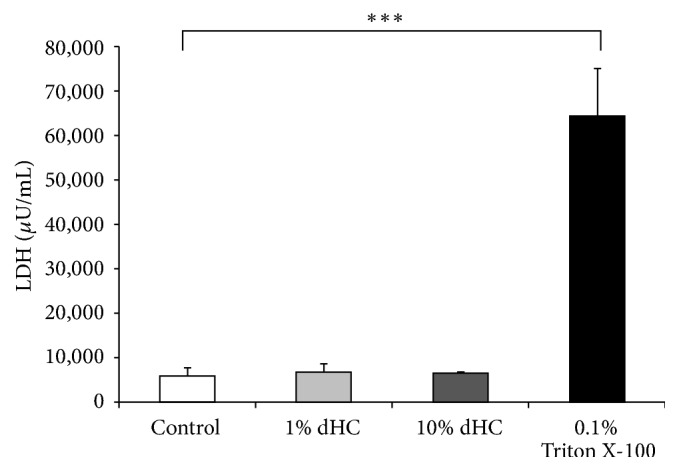
*No cytotoxic effects of dHC on oral keratinocytes*. The cytotoxic effects of dHC on RT-7 cells were assessed by a lactate dehydrogenase (LDH) cytotoxicity assay. 0.1% Triton X-100 treatment and gentle agitation at 25°C for 10 min were used as a positive control. ^*∗∗∗*^Significant differences between the indicated groups at *p* < 0.001 using a Student's *t*-test (*n* = 4).

To investigate the cytotoxicity of dHC on oral keratinocytes, we measured the levels of LDH released from RT-7 cells. As shown in Figure 2, dHC did not exert any cytotoxic effects, up to a concentration of 10%. These results suggest that dHC could be applied to oral care.

### 3.4. Inhibitory Effects of dHC on Chemokine Production by Oral Keratinocytes

**Figure 3:**
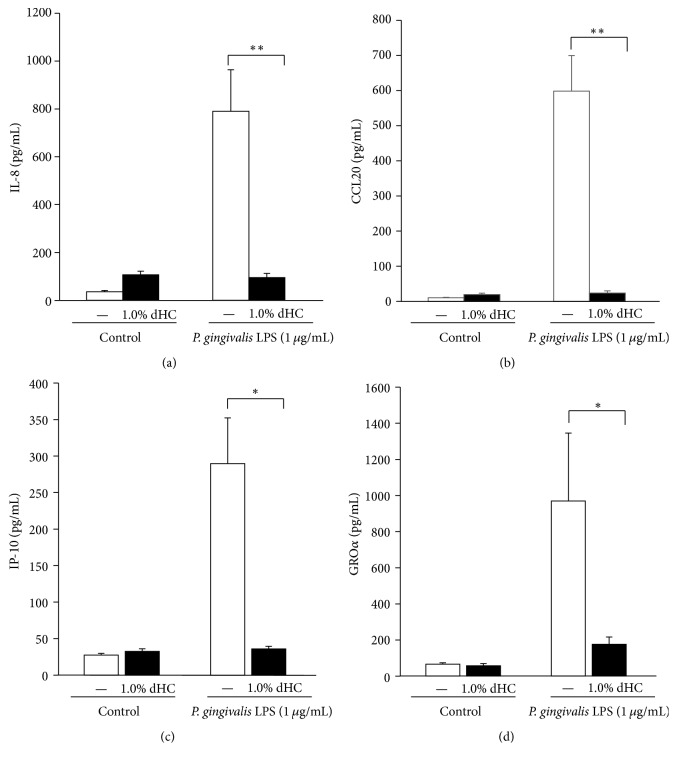
*Inhibitory effects of dHC on chemokine production by P. gingivalis LPS-stimulated oral keratinocytes*. Inhibitory effects of a water decoction of* Houttuynia cordata* (dHC) on IL-8 (a), CCL20 (b), IP-10 (c), and GRO*α* (d) production by oral keratinocytes stimulated with* P. gingivalis* LPS for 24 h. ^*∗∗*^Significant differences between the indicated groups at *p* < 0.01. ^*∗*^Significant differences between the indicated groups at *p* < 0.05 using a Student's *t*-test (*n* = 6).

We have previously demonstrated that CCL20 produced by inflamed gingival epithelial cells appears to be closely connected to the proinflammatory response of the gingiva. This is due to an important regulatory role in specific lymphocyte migration into diseased periodontal tissue [15]. In addition, TLR2, a pattern recognition receptor for LPS from Gram-negative bacteria such as* P. gingivalis*, is strongly expressed in the pocket epithelium of periodontal tissues with chronic periodontitis. This participates in a signaling cascade that upregulates the production of IL-8, IP-10, and GRO*α* [12, 14, 16–22, 45]. We therefore examined whether dHC inhibits the production of IL-8, CCL20, IP-10, and GRO*α* in RT-7 cells stimulated by* P. gingivalis* LPS. We found that 1% dHC significantly inhibited IL-8, CCL20, IP-10, and GRO*α* productions by RT-7 cells stimulated with* P. gingivalis* LPS after 24 h (Figures 3(a), 3(b), 3(c), and 3(d)). This suggests that dHC may be clinically useful as an oral care product to prevent the infectious oral inflammation that is observed during periodontal disease.

### 3.5. Antibiofilm and Anti-Inflammatory Effects of Flavonoid Glycosides

**Figure 4:**
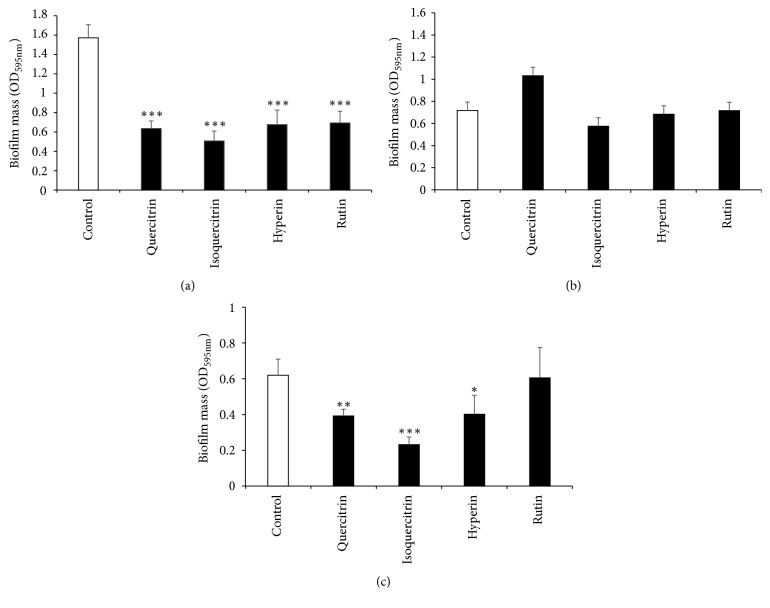
*Antibiofilm effects of flavonoid glycosides on MRSA T31, C. albicans, and F. nucleatum*. Antibiofilm effects of flavonoid glycosides on biofilm formation by MRSA T31 (a), CAD1 (b), and* F*.* nucleatum* (c) at 24-h. A negative control of 2% DMSO was used. ^*∗∗∗*^Significant differences between the indicated groups at *p* < 0.001. ^*∗∗*^Significant differences between the indicated groups at *p* < 0.01. ^*∗*^Significant differences between the indicated groups at *p* < 0.05 using a Student's *t*-test (*n* = 4).

**Figure 5:**
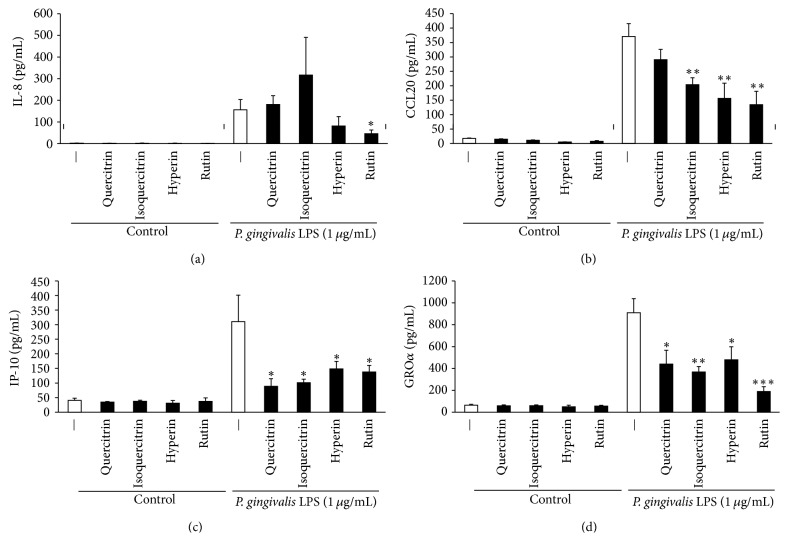
*Anti-inflammatory effects of flavonoid glycosides on chemokine production by P. gingivalis LPS-stimulated oral keratinocytes*. Inhibitory effects of flavonoid glycosides on IL-8 (a), CCL20 (b), IP-10 (c), and GRO*α* (d) productions by oral keratinocytes stimulated with* P. gingivalis* LPS for 24 h. ^*∗∗∗*^Significant differences between the indicated groups at *p* < 0.001. ^*∗∗*^Significant differences between the indicated groups at *p* < 0.01. ^*∗*^Significant differences between the indicated groups at *p* < 0.05 using a Student's *t*-test (*n* = 3).

Previous reports have shown that the leaves of HC contain flavonoid glycosides such as quercitrin, isoquercitrin, hyperin, and rutin [46, 47]. In this study, antibacterial, antibiofilm, and anti-inflammatory assays of four flavonoid glycosides (quercitrin, isoquercitrin, hyperin, and rutin) were performed. As shown in Table 3, these flavonoid glycosides had little antibacterial activities. However, 200 *μ*g/mL of quercitrin, isoquercitrin, and hyperin significantly inhibited 24-h biofilm formation by MRSA T31 and* F. nucleatum* but rutin showed inhibitory effect of biofilm formation only by MRSA T31 (Figures 4(a) and 4(c)). In biofilm formation by* C. albicans*, we could not observe antibiofilm effects by these flavonoid glycosides (Figure 4(b)). Moreover, 50 *μ*g/mL of quercitrin, isoquercitrin, hyperin, and rutin significantly inhibited CCL20, IP-10, and GRO*α* productions by RT-7 cells stimulated with* P. gingivalis* LPS (Figures 5(b), 5(c), and 5(d)). In IL-8 production, suppressive effect was observed only in rutin (Figure 5(a)).

## 4. Discussion

Our study has successfully demonstrated that dHC exerts a moderate antimicrobial effect against microorganisms that normally colonize the oral cavity (Table 2). We have also shown that dHC exhibits antibiofilm effects against MRSA,* F. nucleatum*, and* C. albicans* (Figures 1(a), 1(b), and 1(c)). Finally, we have shown that dHC can inhibit IL-8 and CCL20 production by* P. gingivalis* LPS-stimulated human oral keratinocytes, with no apparent cytotoxic effects (Figures 2, 3(a), 3(b), 3(c), and 3(d)).

Previous studies have reported that medicinal plant extracts can exert moderate antimicrobial effects against oral microorganisms. These include a crude aqueous extract of ripe* Morinda citrifolia* fruit (Indian noni), a methanol extract of* Polygonum cuspidatum*, and a methanol extract of* Syzygium aromaticum* (clove) [23–26]. However, it has been suggested that the use of organic extracts that include ethanol for oral care increases the risks of adverse reactions [35]. Therefore, a water solution of HC poultice ethanol extract (wHCP) used in our study may be safer than the ethanol based extract (eHCP) [36]. Our study has examined the properties of dHC, which is simpler to prepare than wHCP and has been used as health-promoting herbal tea without any reported adverse reactions from our interview survey [34, 36]. Previous studies have demonstrated that medicinal plants also exert antiadherence effects against oral microorganisms [27–29]. In addition to a moderate antimicrobial effect, our results have shown that dHC significantly inhibits adherence after 6 h (Figures 1(a) and 1(b)) and biofilm formation by MRSA and* C. albicans* at 24 h (Figures 1(a) and 1(b)). It also inhibited biofilm formation by* F. nucleatum* (Figure 1(c)).

Finally, HC has also been shown to have an effect on host responses, with previous studies reporting that a 70% ethanol extract of HC dried aerial parts inhibits the production of several inflammatory biomarkers by lung epithelial cells, including IL-6 and nitric oxide (NO), and HC also inhibited lung inflammatory responses in a mouse model of LPS-induced acute lung injury [32]. Furthermore, a HC ethanol extract reduced the production of proinflammatory cytokines through the NF-*κ*B signaling pathway in human mast cells [31]. A water extract of HC has also been shown to exert strong anti-inflammatory effects against* S. aureus* lipoteichoic acid-induced inflammatory responses that are partly attributed to the inhibition of tumor necrosis factor (TNF) expression in dermal fibroblasts [30]. Finally, a powdered extract of HC was recently found to modulate innate oral immune mediators in oral epithelial cells [33], and the mRNA abundance of IL-8 and CCL20 (used as inflammatory mediators) was upregulated in a dose-dependent manner. However, our results demonstrated that dHC inhibited IL-8, CCL20, IP-10, and GRO*α* production by* P. gingivalis* LPS-stimulated human oral keratinocytes. This indicates that dHC can exhibit multiple different activities against microorganisms and host cells and may be useful as an oral care product to prevent infectious oral diseases.

Previous reports have shown that HC contains aldehydes, such as capric aldehyde (decanal), lauryl aldehyde (dodecanal), and decanoyl acetaldehyde (3-oxo-dodecanal, houttuynin), and flavonoid glycosides, such as quercitrin, isoquercitrin, hyperin, and rutin [46–49]. Aldehydes have antibacterial activity; however, the dried leaves of HC could not contain aldehydes because of their instability and volatility. This will be why the antimicrobial effects of dHC were weaker than those of wHCP. Conversely, the antibiofilm and anti-inflammatory effects demonstrated by dHC were similar to those of wHCP. This suggests that the antibiofilm and anti-inflammatory constituents of dHC and wHCP would be flavonoid glycosides. These effects of each flavonoid glycoside are different with bacterial species and cytokines. The use of dHC containing flavonoid glycosides represents a simpler preparation method than wHCP for self-medication.

In Japan, aspiration pneumonia is a serious medical issue in immunocompromised patients, particularly the elderly. We have previously reported a higher prevalence of* Candida* spp.,* P. aeruginosa*, and* Staphylococcus* spp. in the oropharyngeal microflora of patients with cerebrovascular infarction and dysphagia [6]. Reducing adherence and biofilm formation by oral microorganisms can contribute to the prevention of chronic oral infections and potentially severe, systematic opportunistic diseases, particularly in the elderly [9, 10]. Therefore, the use of dHC as herbal tea may strongly contribute to the prevention of aspiration pneumonia.

Povidone iodine, chlorhexidine, and benzethonium chloride are typically used as antiseptic mouthwashes or rinses to prevent oral infections including dental caries and periodontal diseases [50–55]. However, these antiseptics exhibit mucosal cytotoxicity, have a bad flavor, and can cause anaphylactic reactions [54, 56–58]. Therefore, dHC prepared as herbal tea is likely a safer mouthwash than these antiseptics. It also has a milder taste, without odor or cytotoxicity.

The results of our study contribute to the evaluation of dHC as an effective herbal tea that may help prevent infectious oral diseases. Further studies are needed to fully characterize the constituents of dHC and identify the specific factors that exhibit the antibiofilm and anti-inflammatory activities.

## 5. Conclusion

This study demonstrated that dHC exerts a moderate antibacterial effect against MRSA and other microorganisms. It also exhibited antibiofilm effects against MRSA,* F. nucleatum*, and* C. albicans*. We have shown that dHC exerts inhibitory effects on IL-8 and CCL20 production by* P. gingivalis* LPS-stimulated human oral keratinocytes, without cytotoxicity. Our results suggest that dHC has multiple activities in microorganisms and host cells and a herbal tea preparation may be effective in preventing infectious oral diseases.

## Figures and Tables

**Table 1:** Bacterial strains.

Strain	Source
Methicillin-resistant *Staphylococcus aureus* T31	Clinical isolate
Methicillin-resistant *Staphylococcus aureus* COL	Wild type
*Streptococcus mutans* MT8148	Clinical isolate
*Streptococcus mutans* UA159	Clinical isolate
*Streptococcus sobrinus* 1310	Clinical isolate
*Streptococcus gordonii* ATCC10558	Type strain
*Streptococcus oralis* ATCC10557	Type strain
*Streptococcus constellatus* 4528	Clinical isolate
*Streptococcus intermedius* 40138	Clinical isolate
*Streptococcus mitis *JCM 12971	Wild type
*Aggregatibacter actinomycetemcomitans* Y4	Wild type
*Fusobacterium nucleatum *JCM8532	Wild type
*Porphyromonas gingivalis* ATCC33277	Type strain
*Pseudomonas aeruginosa* PAO1	Wild type
*Candida albicans* CAD1	Clinical isolate

**Table 2:** MIC of dHC.

Bacterial strain	MIC	
	(%)^*∗*^	(*μ*g/mL)
Methicillin-resistant *Staphylococcus aureus* T31	5	750
Methicillin-resistant *Staphylococcus aureus* COL	10	1500
*Streptococcus mutans* MT8148	>10	>1500
*Streptococcus mutans* UA159	>10	>1500
*Streptococcus sobrinus* 1310	>10	>1500
*Streptococcus gordonii* ATCC10558	>10	>1500
*Streptococcus oralis* ATCC10557	>10	>1500
*Streptococcus constellatus* 4528	>10	>1500
*Streptococcus intermedius* 40138	10	1500
*Streptococcus mitis* JCM 12921	10	1500
*Aggregatibacter actinomycetemcomitans* Y4	>10	>1500
*Fusobacterium nucleatum *JCM8532	2.5	375
*Porphyromonas gingivalis* ATCC33277	10	1500
*Pseudomonas aeruginosa* PAO1	>10	>1500
*Candida albicans* CAD1	>10	>1500

^*∗*^Concentration of dHC in medium v/v.

**Table 3:** MIC of flavonoid glycosides.

Bacterial strain	MIC (*μ*g/mL)			
	Quercitrin	Isoquercitrin	Hyperin	Rutin
Methicillin-resistant *Staphylococcus aureus* T31	>512	>512	>512	>512
*Candida albicans* CAD1	>512	>512	>512	>512
*Fusobacterium nucleatum *JCM8532	256	512	512	256
